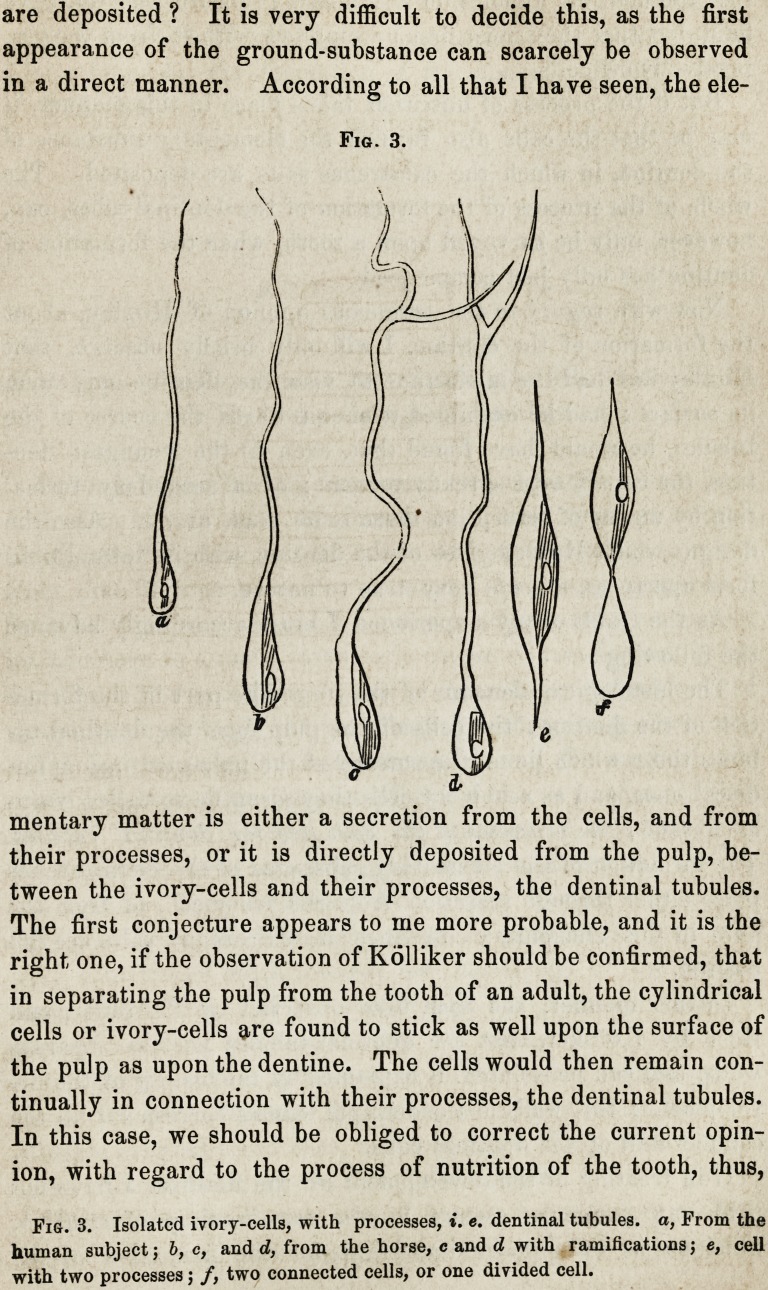# On the Development of the Dentine and Enamel

**Published:** 1857-10

**Authors:** Edward Lent

**Affiliations:** Medical Student of Hamm.


### SELECTED ARTICLES.

### ARTICLE VI.

*On the Development of the Dentine and Enamel.* By Edward Lent, Medical Student of Hamm. Translated from Zeitschrift für Wissenchaftliche Zoologie.

The development of the substance of the teeth is a subject in the history of the development of the tissues of the human body that has already met with a manifold elaboration, and the literature on this subject may be called an important one, since Rashkow, who, in 1835, undertook, under the direction of Purkinje, a minute microscopic examination of it. A treatise recently appeared by Huxley, in the Quarterly Journal of Microscopical Science, vol. i, in which this subject has again been taken up. This essay necessarily excited interest, from the fact of its communicating observations which perfectly contradicted the current opinions concerning the development of the substances of the teeth. I made it my task to examine these observations, and it appeared to me no ungrateful work, to publish in a concise form the results of my investigations, with regard to the development of the dentine and of the enamel, the more particularly, as in so doing, I comply with the request of Professor Kölliker, under whose direction these investigations have been made.

1. *The formation of the dentine.**—The formation of the dentine has always been one of the most obscure points in the development of the teeth; a wide scope was hence given to hypotheses, so that each possibility found its defender, in the history of the development of the teeth. According to the former opinion of authors, the dentine was supposed to grow, by means of gradual layers, upon the germ of the tooth; an opinion which has recently again been taken up in the above mentioned treatise by Huxley.* I shall yet return to this opinion, for the present it may suffice to have mentioned it. The opinion of Rashkow † on the development of the dentine consists in the following :—He supposes a formation of filaments in layers around the pulp, which grow together; between these fibres spaces remain, and these are the dentinal canals or tubes. Since Schwann and Owen, the hypothesis regarding the development of the dentine took another direction. It has been now adopted, that the pulp itself took part in the formation of the dentine, and that the cellular elements of the former have a direct influence in its formation. Schwann thinks ‡ that the round cells of the pulp assume a cylindrical form, melt into each other and ossify. How the dentinal tubules might originate, Schwann does not venture to decide. On the ground of some observations made upon the teeth of pigs, he certainly considered it possible that the cells of the pulp might elongate themselves, and so form the dentinal canals, but being unable to observe in the human teeth that which he had discovered in the teeth of pigs, he gave up this opinion. Henle § supposed that the cells of the pulp participated in such a manner in the formation of the dentine, that the cells form the fundamental substance, that the nuclei elongate, unite themselves, and so form the dentinal tubules. Owen || holds a similar opinion. In the cells of the pulp, which he calls mother-globules, he considers there are nuclei and secondary cells, which coalesce, and thus form the dentinal tubules, whilst the basic substance is formed by the mother-globules. Tomes'¶ opinion is similar to that of Henle, in adopting cells and a secondary substance upon the surface of the pulp. According to him, the latter is the ground sub-

* On the Development of the Teeth, and on the nature and import of Nasmyth's Persistent Capsule, by Thomas H. Huxley, F. R. S.

† Meletamata circa Mammalium Dentium Evolutionem Dissertatio inauguralis, Vratislaviæ, 1835.

‡ Mikrokopische Untersuchungen, Berlin, 1839.

§ Allgemeine Anatomie, Leipzig, 1841.

|| Odontographie, &c., London, 1840-'45.

¶ A Course of Lectures on Dental Physiology and Surgery, London, 1848.

stance, and the cells range themselves upon each other to form the dentinal tubules. The nuclei of the same are the proper cavities of the dentinal canals. Kölliker * says, that the basic substance of the dentine originates from the superficial cylindrical cells of the tooth-pulp, which he called ivory cells, and in fact, only from these; these multiply, coalesce and ossify. The dentinal tubules seemed to him to be the remains of the cellular cavities, whose borders became more consolidated. Marcusen † pronounces no opinion upon this point, and has reserved it for future investigations, but he seems to consider the dentinal tubules rather identical with bone corpuscules. According to him, the membrana præformativa grows first into bone, and then the pulp gets also metamorphosed into bony substance. The more recent opinion of Huxley I have already mentioned above.

Among these different hypotheses, for as such most of the above mentioned opinions must be designated, an important difference may be reasonably observed, so that two strictly separate opinions would seem to be entertained; whilst some of them allow the cellular elements of the pulp to take part in the formation of the dentine, others contend that they do not. Although the assumption that the histological elements of the pulp had no direct connection with the formation of the dentine, originated with older authors, who speak but of calcareous deposits; and although the same is simply mentioned by later authors, I was, nevertheless, obliged to draw attention to that difference, because Huxley set himself up as a defender of the older opinion. Huxley maintains that, between the membrana præformativa, which covers the pulp of the tooth and the pulp itself, a light deposit grows without any structure at first. This having acquired a thickness of $\frac{1}{5000}$th of an inch, takes a spotted appearance, whilst on the surface very numerous, but very small, cavities show themselves. According to him, these funnel-shaped cavities, which enter into the calcific deposit, are the dentinal tubules. According to this view, the tubes arise

* Mikrokopische Anatomie, vol. ii, part ii, section 1.

† Ueber die Entwicklung der Zähne der Säugethiere, St. Petersburg, 1850.

secondarily, by means of resorption, but this is founded, as I will show subsequently, upon a false interpretation of a microscopical object. It seems, however, as if Rashkow also sided with the opinion that the histological elements of the pulp took no part in the formation of the dentine. Rashkow speaks of filaments which grow around the pulp; but whether these filaments are in connection with the elements of the pulp, and if so, how connected, he does not state. The connection between the pulp and the formation of dentine, he explains in the obscure phrase, "Germinis dentalis parenchymate materiam suppeditante." Now all the remaining authors since Schwann, are decidedly opposed to the older authors, and Huxley, do not doubt but that the cellular elements of the pulp participate in the formation of the dentine, and the differences in their diverse opinions are only founded upon the different construction of the origin of the canals of the tooth. The following conjectures may then be advanced or suggested: The walls of the cells become thickened, through calcareous deposits, the hollow of the cell is filled up, leaving a canal (the nucleus,) which remains open. This canal is the dentinal canal; naturally several cells participate in the formation of a dentinal tubule. This opinion is considered, by Kölliker for instance, as probable. The cells form the dentinal tubules in such a manner, that their walls turn into those of the tubules. The nuclei of cells elongating and fusing into each other, form the walls of the dentine.* Around them the secretion of lime takes place; such is the opinion of Henle, and partly also of Tomes. The deposition of lime takes place around them. Schwann and Kölliker thought of this possibility, but gave up the idea. According to my observations, I must consider this latter opinion as the correct one, and I will now enumerate the results of the same in succession.

The teeth of which I availed myself for my investigations, were chiefly human teeth, from new born infants, and from the

* Does not this mean that the nuclei become the canals, and the cell-walls the walls of the canals? If so, this is the opinion of Tomes. Is not the nucleus of the dentinal cell the analogue of the nucleus of the lacunal cell of bone, and is not this that which Lent means?

fœtus of six months, but I have also made use of the embryo or uncut teeth of the calf, of the rabbit and the squirrel, and, subsequently, of the new-born horse.

Schwann had already observed, that, if in embryonal teeth, the tooth is drawn out of the top cap, a quantity of cylindrical cells remain upon the young tooth, indeed, similar to those found upon the surface of the pulp. He had also seen in the teeth of pigs, that those cylindrical cells resting on the pulp, ended in subtle fibres, which he could not find in those of men; Kölliker, however, succeeded in doing so. On the teeth of calves, which had not yet been cut, and also on the teeth of the human embryo, I have found the same. If such teeth are macerated for some time in diluted muriatic acid, and if then the young tooth, setting like a cap upon the pulp, is separated, then, by means of microscopic examination, these cylindrical cells are to be seen on the surface of the pulp, ending in filaments, and they represent a figure as if the pulp was surrounded by a wreath of bristles. They are the cells which Kölliker has already depicted in his Microscopical Anatomie, fig. 209, with regard to which I have only to observe, that the cells represented there, partially admit of the supposition that the processes are not the continuation of the cellular walls, which, however, is the case. That these filaments stand in some close connection with the dentinal tubules, was a very near or close conjecture. It was now important to ascertain whether these filaments could not be pursued further into the dentine. I macerated young calves' teeth in muriatic acid until the dentine was so soft that it could easily be pierced by a needle. I must observe here, that this maceration is not to be carried too far, as with too lengthy a treatment, the elements dissolve too much. From this softened dentine, I placed part of the pulp, with a piece of dentine cleaving to it, under the microscope, and it already appeared to me, as if the processes of the cylindrical cells, close to the dentine, could be traced into it; the same object was then carefully pulled asunder, and now it showed itself that the whole of the dentinal tubules could be isolated as processes of the cells. They represent themselves as they are delineated in fig. 3, *a* to *d*. *a* is a cell from a human tooth, *b*, *c*, and *d*, from the horse. The cells of the calf are similar to the human cell. Altogether, I have found that, their size excepted, these cells were alike in several animals. As I have already observed, I first discovered those cells upon the teeth of calves, and I then sought for the same result upon the human teeth ; here I easily succeeded in arriving at the same result, by means of the same treatment. Huxley had seen but once, that a process of a cell had extended into the dentine, and seems to consider that only as an accident. It is altogether remarkable that he wholly ignores the already mentioned observation of Schwann, upon the teeth of pigs, and that of Kölliker upon the human teeth.

A certain amount of good fortune is required, to make use of the macerated teeth, just at the time, when the calcareous salts are sufficiently soaked out, so that the isolation of the dentinal tubules may be easily executed, because if the dentine is yet too solid, the processes of the cells will tear off because the dentine will not let them loose; if the dentine is too soft, the whole of the tissue is so brittle, that destroyed masses only are seen; and pressure upon the compressorium immediately destroys the object. On account of the difficulty of isolation, in consequence of the processes tearing away from the cells, a large quantity of cells are seen, which show distinctly that they have lost their processes, and also, on the other hand, a multitude of processes or dentinal tubules, which are separated from the cells. These cells might be 0.01—0.02‴ long; the elongation of the processes mostly takes place by degrees; the long processes show the diameter of a dentinal canal, consequently the centre is 0.001‴. The cells often show their nucleus, still, if this is indistinct, an addition of acetic acid will make it lighter, or by means of a diluted solution of iodine it will color itself intensely yellow. The cells have besides usually a granulated substance, which appears all the more granulous and dark, the more the acid has operated. I even succeeded in seeing in some cells distinct ramifications upon the processes, like in fig 3, *c* and *d*. It was but of late that I had at my disposal the head of a new-born horse, and I found upon its grinders these cells with the most beautiful ramifying processes. These processes were easily seen to form tubes, which here and there showed also to possess some contents. I will yet mention here, that when I subsequently for other purposes, treated the teeth with diluted sulphuric acid, or with nitric acid, or also with concentrated acid of vinegar, I got these cells even more beautiful than by means of muriatic acid, at least with the first mentioned acids; the acid of vinegar does not affect the dentine enough.

The question now arises, which cells of the pulp enter into this state of formation of processes? The best explanation of this is given by the young tooth, at the period when the formation of the dentine ought just to commence. When there is not any trace of dentine yet the pulp shows in its whole mass the construction, as is generally described. It consists of a chiefly granular, and somewhat fibrous ground-substance, in which are imbedded cells and nuclei of cells, mostly of a roundish shape; at the commencement of calcification only, vessels and nerves develop themselves in it. The pulp is covered all over by the membrana præformativa, which, however, really is a membrane, and not as Marcusen supposed, the boundary layer of the connective tissue of the pulp. By maceration in acid of vinegar or kali, it separates itself in a bulk, it can also be produced in pieces, by being pulled off from a tooth-pulp, and the still abundantly adhering cells, &c., being destroyed through the operation of alkalies. Now at the spot only where the dentine shall develop itself, the cells of the pulp elongate themselves, and get a cylindrical shape, and only now it has the appearance, as if the pulp was covered by a cylindrical epithelium. This bed of cells, resembling a cylindrical epithelium, has received, by Kölliker, the name of ivory membrane, membrana eboris; the individual cells he has described as ivory cells. These names may be justified, as they denote the purpose of the structures; this membrane must, however, not be isolated, and it may also be observed, that the whole pulp is not covered with these cylindrical ivory-cells, as is supposed by some, but only that part which is just proceeding to the formation of dentine, so that the cells which lie on the top of the pulp, become first cylindrical, and this process of transformation progresses from above downwards, until finally, nearly the whole pulp is covered with such cells. When a cell has received a cylindrical form, it shoots forth its process, which goes on to elongate, until it has received its above specified length (compare fig. 1.) Now does *one* cell suffice for the formation of a dentinal tubule, or do, perhaps, two or more unite for that purpose? It seems to be the rule, that one cell forms one den-

Fig. 1.

tinal tubule, but that also two (or more ?) cells may unite; the following facts avouch this: (1) cells are observed, which shoot

Fig. 1. Section of the point of a human fœtal grinder (cheek tooth,) upon which the formation of the dentine and of the enamel has recently commenced. *a,* Pulp or germ of the tooth, with vessels; *b,* so called ivory membranes, consisting of ivory cells; *c,* complete ivory; *d,* complete enamel; *e,* membrana præformativa; *é* membrana præformativa (or Huxley membrane) after maceration in acid of vinegar.

Fig. 4. A particle of the membrana præformativa from the tooth of a young horse, with the impressions derived from the fibres of the enamel.

forth processes on both sides (fig. 3 *e;* and (2) connections of two cells are seen, in such a manner, that it appears as if one cell had received a contraction (fig. 3 *f*,) both which forms, Kölliker has already depicted (in fig. 209 of his Mikroskop.

Fig. 2.

Anatomie.) It also happens, that two and more nuclei are seen in cylindrical cells; we could then also suppose, with Kölliker, that these cells received, by means of a process of division, the property of forming the long dentinal tubes; but it seems, that in many cases, one cell, in fact, suffices to produce the whole of a dentinal canal, which is only possible, if it is richly nourished from the pulp. But these variations in the process of formation do not affect in the least the main subject, that the ivory cells form the dentinal canals. Besides, as soon as the formation of the processes begins, calcareous layers show themselves at the same time. How originates, then, the fundamental substance of the dentine, in which the calcareous salts

Fig. 2. Complete enamel, to which the membrane of the enamel remained adherent. *a*, Membrane of the enamel, consisting of cells of enamel; *b*, complete prisms of enamel; *c*, membrana præformativa (Huxley membrane,) visible after maceration in vinegar.

are deposited? It is very difficult to decide this, as the first appearance of the ground-substance can scarcely be observed in a direct manner. According to all that I have seen, the ele-

Fig. 3.

mentary matter is either a secretion from the cells, and from their processes, or it is directly deposited from the pulp, between the ivory-cells and their processes, the dentinal tubules. The first conjecture appears to me more probable, and it is the right one, if the observation of Kölliker should be confirmed, that in separating the pulp from the tooth of an adult, the cylindrical cells or ivory-cells are found to stick as well upon the surface of the pulp as upon the dentine. The cells would then remain continually in connection with their processes, the dentinal tubules. In this case, we should be obliged to correct the current opinion, with regard to the process of nutrition of the tooth, thus,

Fig. 3. Isolated ivory-cells, with processes, *i. e.* dentinal tubules. *a,* From the human subject; *b, c,* and *d,* from the horse, *c* and *d* with ramifications; *e,* cell with two processes; *f,* two connected cells, or one divided cell.

that the dentinal tubules do not directly absorb the nutritive fluid, but that they must first be supplied with it from the respective ivory-cells.

But if the process of nutrition takes place in this manner, it may be that the cells also furnish the elementary substance of the dentine, in which the calcareous salts are deposited. The whole of the process of the formation of the dentinal tubes, can, however, only be surveyed upon a tooth, when the formation of dentine has only just commenced.

Now with regard to the erroneous opinion of Huxley, about the formation of the dentine, I will only briefly observe, that Huxley has had the misfortune to view the dentine only upon its surface; had he examined it once towards the course of the tubules, he would have found that, even in the youngest dentine, the canals were already present; of a secondary formation by means of resorption there is no trace at all. Also the designs which Huxley gives of the dentine, with its infundibuliform apertures, are not very true to nature.

As the result of my experience, I must accordingly advance the following:

The histological elements of the pulp take part in the formation of the dentine; the cells of the pulp form the dentinal tubules those which lie on the surface of the pulp turn into cylindrical ones, and as real ivory-cells they form the so-called ivory-membrane; *the processes of the cells are the dentinal tubules.* The formation of the elementary substance of the dentine takes place either through secretion from the ivory-cell, or from the pulp in this elementary substance the calcareous salts are deposited.

In this manner, the fact, first observed by T. Müller, and then by Kölliker, that the dentinal tubules possess separate walls, and can be isolated, is explained by the history of the development; the walls of the dentinal canals correspond to the cellular wall of the dentinal cells.

If in treating of the development of the dentine, I have not paid any particular regard to the membrana præformativa, which seems to be more important, for the formation of the tooth than has has hitherto been assumed, I will resume the subject, in which I shall briefly communicate the results which I have found on the formation of the enamel.—*British Jour. of Den. Sci.*

## Figures and Tables

**Figure f1:**
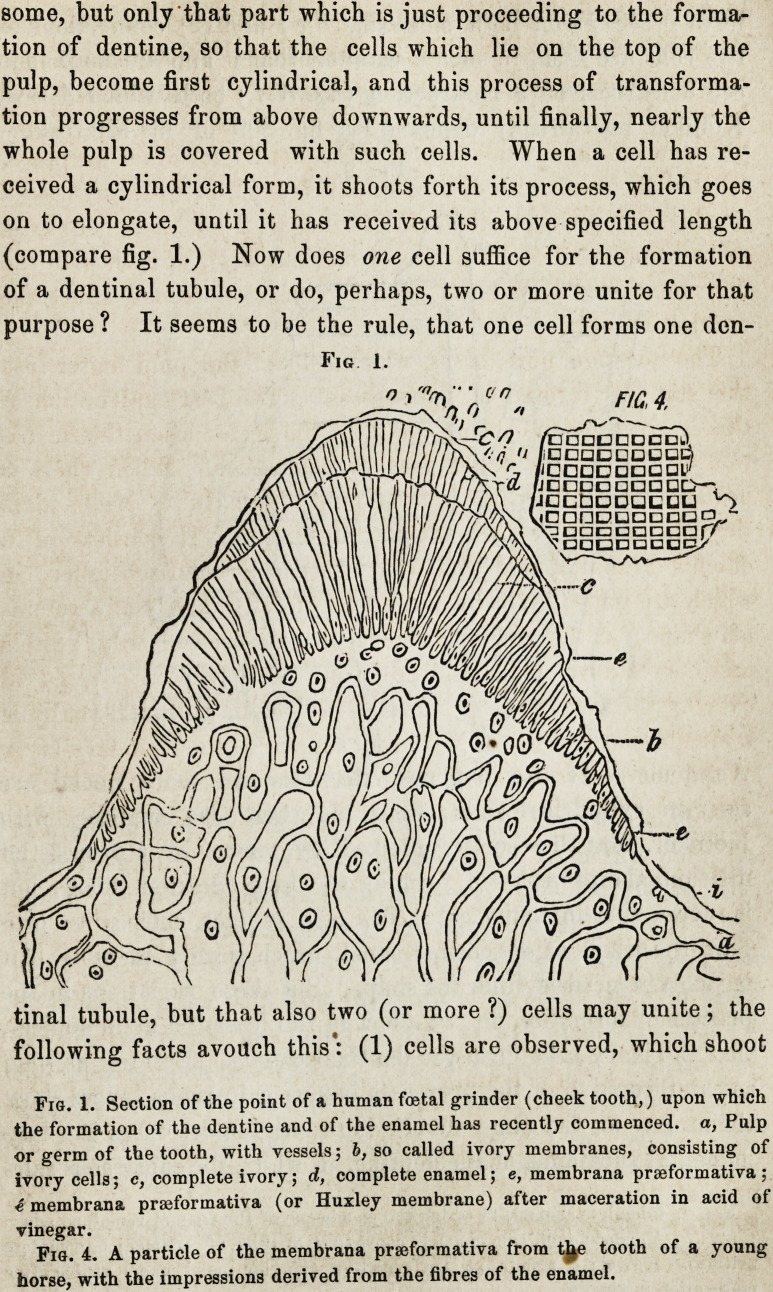


**Fig. 2. f2:**
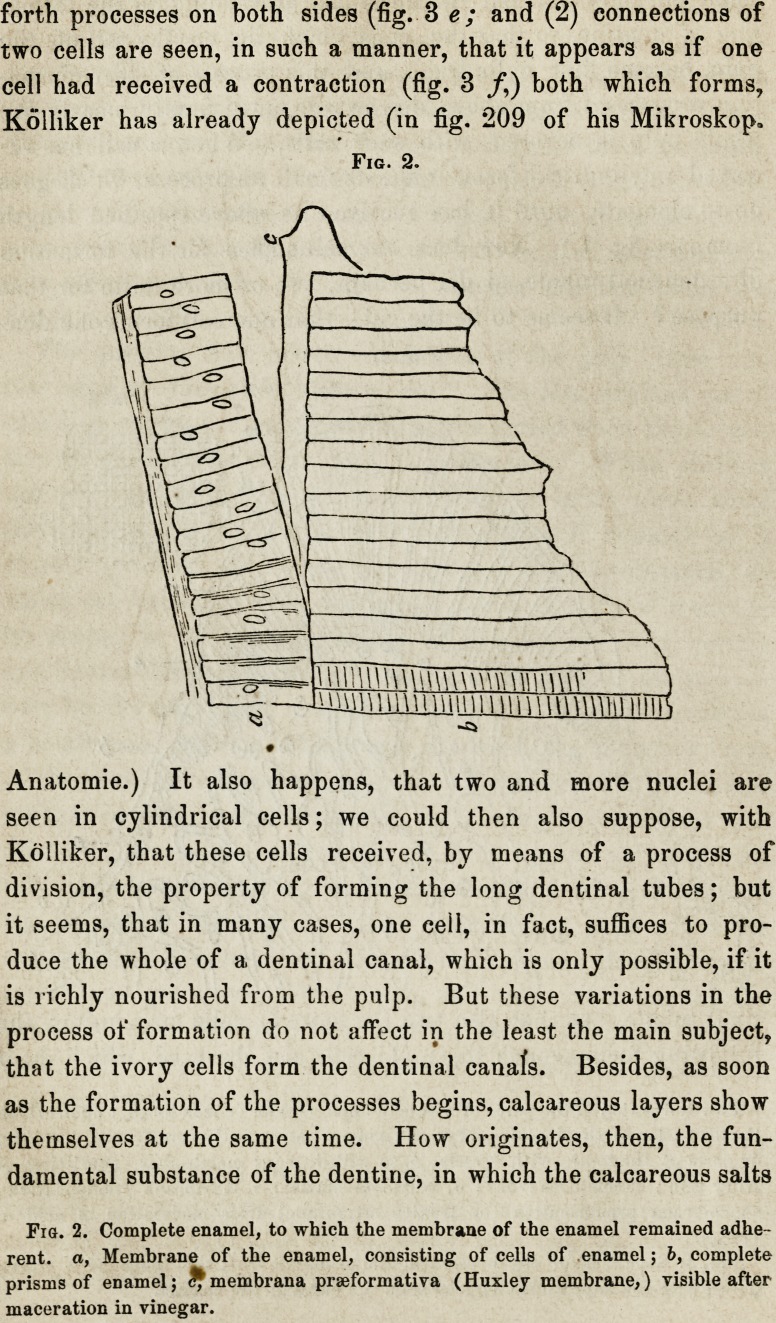


**Fig. 3. f3:**